# A case study on the impact of Ramadan on biomechanical and physiological markers in a female collegiate student-athlete

**DOI:** 10.3389/fspor.2025.1576424

**Published:** 2025-10-21

**Authors:** Joseph Amitrano, Tariq Ameer, Gina Lewandowski, Lauren Calabrese, Dhruv R. Seshadri

**Affiliations:** ^1^ Department of Bioengineering, Lehigh University, Bethlehem, PA, United States; ^2^ Department of Multicultural Affairs, Lehigh University, Bethlehem, PA, United States; ^3^ Department of Athletics, Lehigh University, Bethlehem, PA, United States

**Keywords:** wearable technology, women's health, human performance, Ramadan, sports medicine

## Abstract

This case study investigates the impact of Ramadan, a month of fasting and prayer, on a single female Division I collegiate athlete leveraging wearable technologies, Beyond Pulse and Whoop 4.0, and subjective questionnaires to collectively monitor biomechanical, physiological, and psychological changes before, during, and after Ramadan. Beyond Pulse monitors performance metrics, including distance, speed, and heart rate (HR), while the Whoop 4.0 focuses on physiological data such as HR, heart rate variability (HRV), sleep quality (including sleep duration and debt), and exertion metrics like strain and recovery. Subjective measures, including rating of perceived exertion (RPE), stress, and energy levels, were recorded daily to monitor load-response following training. Analyses revealed a 14.6% reduction in sleep performance during Ramadan and persisting post-Ramadan. Stress, muscle soreness, and energy all changed during Ramadan (*p* = 2.00 × 10^−6^; *p* = 0.001; *p* = 0.0058, respectively), with stress remaining elevated post-Ramadan (*p* = 0.028), likely driven by exams and disrupted circadian rhythm. Team-level analysis shows declines in distance during Ramadan (*p* = 9.00 × 10^−4^) and rebound afterward (*p* = 3.00 × 10^−5^), alongside shifts in workload (*p* = 0.024) and training impulse (TRIMP, *p* = 0.014), offering a baseline to distinguish from periodization of training and effects of Ramadan. These results illustrate how Ramadan combined with academic demands substantially effects both physiological and psychological well-being in a Division 1 female athlete. This case study, to our knowledge, is the first to use wearable technology and subjective measures to track Ramadan-related changes in a division 1 collegiate female athlete, offering insights into holistic monitoring.

## 1 Introduction

Ramadan, a 29–30 day lunar month observed by Muslims worldwide, marked by stringent practice of fasting from sunrise to sunset (1, 2). During this period, healthy Muslims abstain from food and drink during daylight hours (10–18 h), fostering reflection and communal solidarity (3). Each day begins with a pre-dawn meal (Suhoor), and ends with an evening meal (Iftar) to break the fast followed by nightly prayers (Taraweeh) (2, 4–7). Consequently, Ramadan can influence sleep patterns and dietary habits, affecting individuals holistically (2).

Athletes who observe Ramadan encounter unique challenges, balancing training and competitive schedules with religious requirements. The reduced caloric intake during Ramadan can significantly impact an athlete's performance, leading to changes in mood and body composition such as reductions in body weight and BMI (3). The changes in diet composition, along with late evening meals, late prayers, and early breakfasts, can lead to behavioral modifications, altered sleep patterns, and increased fatigue (2, 6). Nonetheless, athletes can maintain performance during observation with proper planning and strategies.

Athletes observing Ramadan are typically monitored through a combination of subjective and objective measures to track performance and health (1, 8–12). Despite these efforts, research on continuous monitoring of athletes and the relationship between observation and injury risk remains limited. Preliminary evidence indicates that fasting may heighten susceptibility to muscle fatigue and dehydration, potentially increasing the risk of soft-tissue injury.

Literature suggests Ramadan's impact on athletic performance encompasses various physiological, psychological, and behavioral adaptations. Souissi et al. leveraged Wingate anaerobic tests to demonstrate reductions in peak power output and increased fatigue during Ramadan, which was largely due to sleep disruptions and altered glycogen stores (13). Similarly, Chaouachi et al. studied elite judokas and found no significant changes in individual sprint performance but observed minor declines in repeated effort performance, likely due to fatigue accumulation and dehydration during fasting, highlighting the interplay between hydration and fatigue management (14).

In aerobic performance, Brisswalter et al. investigated the effects of Ramadan fasting on 5,000-m running performance, documenting a 5% reduction in performance (identified by running times). This performance decline was attributed to physiological changes induced by fasting, particularly in muscle strength and oxygen uptake kinetics. The study also found a significant decrease in Maximal Voluntary Contraction by 3.2%, indicating a reduction in muscular strength, which is crucial for maintaining performance over middle-distance races. Additionally, there was a notable 51% increase in the time constant oxygen kinetics, pointing to slower and less efficient aerobic energy production. This suggests a greater reliance on anaerobic energy pathways during the run, leading to quicker fatigue (15). Aziz et al. explored the effects of Ramadan on endurance, incorporating shuttle-run tests over time. An initial decrease in performance during Ramadan was observed: 5,448 ± 845 m during fasting vs. 5,649 ± 715 m in a non-fasted state (*p* = 0.023). However, the authors suggested that performance stabilized as the month progressed, indicating an adaptation to fasting with tailored training. This adaptation underscores the resilience athletes can develop with appropriate adjustments to their regimen during fasting periods (5). Furthermore, Bouhlel et al. conducted a study on rugby players and observed a notable shift in energy substrate (carbohydrates and fats) utilization during moderate-intensity exercise throughout Ramadan. The athletes exhibited an increased reliance on lipid oxidation and a corresponding decrease in carbohydrate utilization, likely influenced by the decreased glycogen stores and overall energy intake associated with fasting. Specifically, the study highlighted an increase in the lipid oxidation rate from a control value of 199.1 ± 20 mg/min to 265 ± 38 mg/min by the end of Ramadan. This adaptation highlights the body's capacity to adjust its energy utilization strategies to maintain performance under altered nutritional conditions (16).

The impact of Ramadan on sleep is well documented where changes in sleep patterns during the month can diminish performance (17). Chamari et al. assessed top-level Tunisian soccer players over 2-years. The authors demonstrated a reduction in total sleep time and poorer sleep quality during Ramadan (18). Similarly, Kerkeni et al. used actigraphy to examine sleep-wake patterns in semi-professional student-athletes and found significant reductions in sleep duration, dropping from about 7 h before Ramadan to 5 h during the second half of the month (19). They also reported delayed sleep onset by approximately 2.5 h, poor sleep quality, increased insomnia symptoms, and excessive daytime sleepiness (19). These findings highlight the substantial disruptions in sleep experienced by athletes during Ramadan, especially when balancing academic and athletic demands. Though these studies provide valuable insight into adaptation during Ramadan, there lacks continuous objective metrics, particularly when assessing athletes and their performance.

Wearable technology has emerged as a tool to address this gap, enabling continuous monitoring of physiological parameters. Alghamdi et al. utilized Fitbit devices to analyze changes in physical activity and sleep patterns among 36 Type 2 diabetes patients during and after Ramadan. Participants wore the Fitbits continuously for seven days in each period, revealing a notable decrease in total sleep duration during Ramadan, with median daily sleeping hours recorded at 6.03 h compared to 7.02 h post-Ramadan. Despite this reduction in sleep, the study found no significant change in physical activity levels between the two periods (20). This study underscores the potential of wearables in providing real-time data and actionable insights, paving the way for tailored strategies to support athletes during Ramadan.

Expanding research with advanced methodologies will provide deeper insights into managing performance and health during Ramadan. Wearable technology has become a tool for athletes and coaches, providing real-time data on various physiological and biomechanical parameters to optimize performance, recovery, and nutrition (8, 21–24). These devices enable precise monitoring of factors such as body composition, sleep patterns, and performance, especially during lifestyle changes like fasting. By leveraging advanced sensors and algorithms, athletes can develop tailored strategies to maintain peak performance, enhance recovery, and reduce the risk of injury (25).

Another avenue of assessment is utilization of subjective assessments and scores. These metrics are reported by the athlete as a response to training or competition stimulus. Subjective reporting can be an additional source for identifying potential issues (10). For example, consistent reports of high rate of perceived exertion (RPE) or muscle soreness may signal overtraining (26, 27). On the other hand, consistent reports of low RPE and low soreness may indicate undertraining, also indicating injury risk (28). Early detection of these metrics enables interventions, such as modifying training intensity, incorporating additional recovery strategies, or providing psychological support, thereby maintaining performance and health (21, 22, 28, 29). By incorporating both subjective and objective measures, a combined approach would facilitate a holistic assessment, allowing adjustments to training loads, enhance recovery, and address psychological factors, safeguarding the health and performance of athletes during Ramadan (10, 29, 30).

This case study examines the application of wearable technology in sports to provide real-time data and actionable insights, focusing on optimizing athlete performance during periods of external challenges, such as Ramadan. The aim is to enable athletes to balance the physical and physiological demands of training and competition while accommodating the effects of religious practices to maintain peak performance. The case study presents, to our knowledge, the first example of monitoring a division 1 female collegiate athlete over this period to understand the interplay between scholastic and on field rigors faced by a student athlete during a critical period.

## 2 Materials and methods

Participants were given informed consent prior to the start of the study. The study was conducted in accordance with the Declaration of Helsinki and approved by the Institutional Review Board of Lehigh University (2113291-6, 12/19/2023). This IRB-approved case study conducted a comparative analysis of biomechanical data obtained from the Beyond Pulse monitor, physiological metrics gathered from the Whoop 4.0, and subjective reports from a single Division I female soccer athlete observing Ramadan (20 years old), benchmarked against 19 other non-Ramadan observing team members. The study spanned the Spring 2024 season, from February 11th to April 29th. Data collection occurred during every session at university facilities using the Beyond Pulse workload monitor, Whoop 4.0, and subjective data through questionnaires.

### 2.1 Data collection

**Figure 1:**
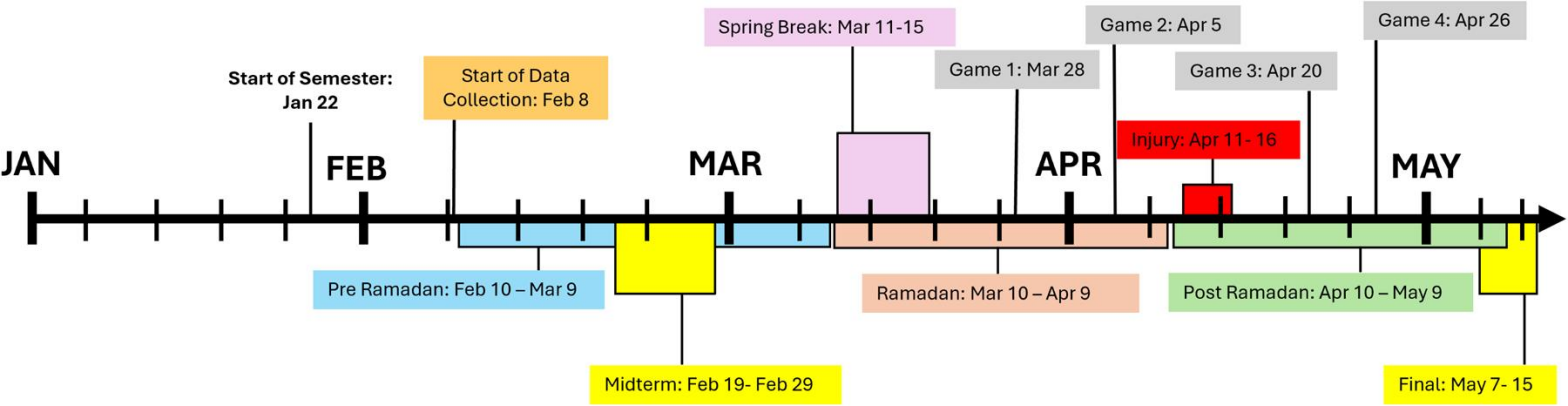
Timeline illustrating key events in the academic and athletic schedule for the Spring 2024 semester. The timeline spans from the start of the semester on January 22 to its conclusion on May 15. Key academic milestones include the pre-Ramadan period (February 10–March 9), midterms (February 19–February 29), Ramadan (March 10–April 9), post-Ramadan (April 10–May 9), and finals (May 7–May 15). Significant athletic events are marked by four games occurring on March 28, April 5, April 20, and April 26, alongside a specified injury period from April 11 to April 16.

To collect training and game data, the Beyond Pulse workload monitor (Beyond Pulse, OR) was employed. Training sessions were conducted four days per week throughout the season, with games on March 28th, April 5th, April 20th, and April 26th (Figure 1). The Beyond Pulse device, a chest-worn strap, incorporates a 2-lead ECG (1 Hz) for heart rate monitoring and an inertial measurement unit (IMU) with an accelerometer for biomechanical analysis (100 hz). The Beyond Pulse Coaching App (Beyond Pulse, 2015) was utilized to initiate, terminate, and save sessions containing data.

To collect physiological data the Whoop 4.0 fitness tracker (Whoop, 2012) was worn continuously for the duration of the study. The Whoop 4.0, a wrist-worn device, utilizes photoplethysmography (PPG) to continuously monitor physiological metrics (52 Hz) and generates a daily average. Data was uploaded to the Whoop Unite Cloud Database (Whoop, 2012) daily, where it was saved, then manually synchronized with biomechanical data for analysis.

Subjective data was collected through two questionnaires administered daily via Google Forms (Google Forms, 2008). The first questionnaire was completed in the morning around 7 am to assess readiness (Supplementary Table S2). The second questionnaire was filled out immediately after athletic activity to provide a post-activity assessment (Supplementary Table S3). These questionnaires evaluated RPE (Borg Scale: 6–20), stress levels (1–10), muscle soreness (6–20), and sleep quality (1–10. The data was stored in CSV files and imported for analysis.

### 2.2 Data analysis

All analysis was performed in Python version 3.10. Raw data was imported, cleaned, and names were standardized via fuzzy matching, allowing each observation to be summarized at three levels: “Player”, “Position”, and “Team”. Each observation was assigned to Pre-Ramadan (before March 10), Ramadan (March 10–April 8), or Post-Ramadan (after April 8). For each metric we computed the period-specific mean and standard deviation. Prior to hypothesis testing, we assessed normality and homogeneity of variances; if all period samples were approximately normal and variances equal, a one-way ANOVA was applied, otherwise a Kruskal–Wallis rank-sum test was used. When the omnibus *p*-value was below 0.05, we performed either independent *t*-tests following ANOVA or Mann–Whitney *U* tests following Kruskal–Wallis, applying a Bonferroni correction. Statistical significance was annotated at *p < 0.05, **p < 0.01, and ***p < 0.005. The Training Efficiency Index (TEI), integrates internal and external training load metrics to assess how effectively an athlete translates training efforts into physiological adaptations was computed based on distance covered as the external workload and Edward's TRIMP as the internal metric (31).

Edward's TRIMP is calculated using the formula:

TRIMP=∑(DurationinHRZone×IntensityFactor)

where the intensity factor corresponds to heart rate zones:

Zone 0%–50% of max HR: Intensity Factor = 1

Zone 50%–60% of max HR: Intensity Factor = 1

Zone 60%–70% of max HR: Intensity Factor = 2

Zone 70%–80% of max HR: Intensity Factor = 3

Zone 80%–90% of max HR: Intensity Factor = 4

Zone 90%+ of max HR: Intensity Factor = 5.

TEI is calculated using the formula:

$$\mathrm{TEI}=\frac{\mathrm{External}\;\mathrm{Load}\;(\mathrm{distance}\;\mathrm{covered})}{\mathrm{Internal}\;\mathrm{Load}\;(\mathrm{Edwar}d^{\prime }s\;\mathrm{TRIMP})\;}$$

A graphical representation of the TEI was generated (Supplementary Figure S2), illustrating daily values with a 3-day moving average for both the team and the individual athlete. Additionally, bar graphs were created to highlight differences in the athlete's performance metrics.

## 3 Results

**Figure 2:**
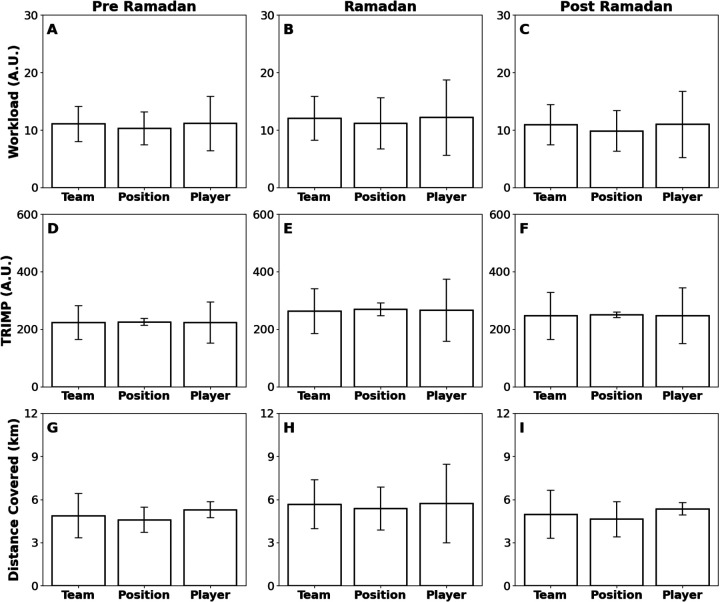
Bar plots displaying three performance metrics—Workload (A.U.), Training Impulse (TRIMP) (A.U.), and Distance Covered (km)—across three analytical dimensions: Team, Position, and Player. Each row presents a distinct metric measured or derived from the wearable device Beyond Pulse. Panels **(A–C)** depict Workload, panels **(D–F)** show TRIMP, and panels **(G–I)** illustrate Distance Covered. The subfigures in each row compare metrics within different contexts: Team-level (first column), Position-level (second column), and Player-level (third column). Each subfigure in the row corresponds to specific periods: Pre-Ramadan **(A,D,G)**, Ramadan **(B,E,H)**, and Post-Ramadan **(C,F,I)**. Statistically significant differences are denoted by asterisks (*p < 0.05; **p < 0.01; ***p < 0.005) with effect sizes (r: non-parametric, d: parametric) denoted; comparisons without asterisks were not significant.

**Figure 3:**
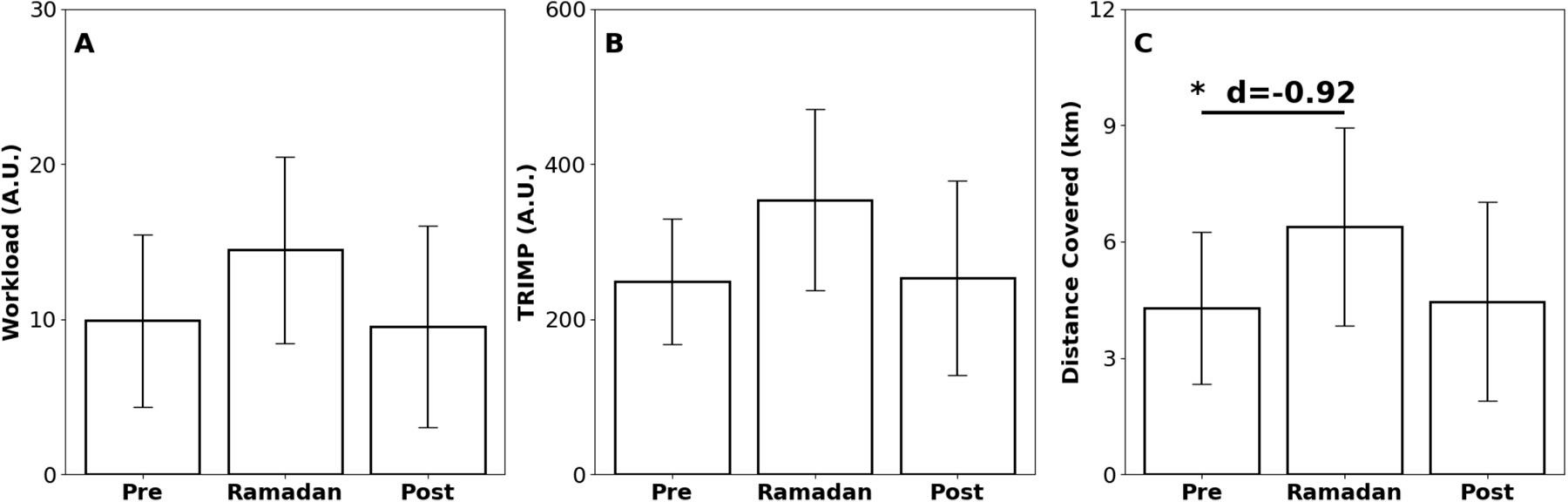
Bar plots showing three performance metrics for the individual player—Workload (A.U.), TRIMP (A.U.), and distance covered (m)—across three time periods: Pre-Ramadan, Ramadan, and Post-Ramadan. The data is measured or derived from the wearable device Beyond Pulse. **(A)** Workload, **(B)** training impulse (TRIMP), **(C)** Distance Covered. Statistically significant differences are denoted by asterisks (*p < 0.05; **p < 0.01; ***p < 0.005) with effect sizes (r: non-parametric, d: parametric) denoted; comparisons without asterisks were not significant.

This case study presents an examination of the impact of Ramadan on the performance and physiological metrics of a Division I female soccer athlete, compared to their non-observing teammates. Analysis of biomechanical data from the Beyond Pulse workload monitor (21,196 data points) consisted of a team-, position-, and player-level performance across the Pre-Ramadan, Ramadan, and Post-Ramadan periods (Figures 2, 3 and Table 1, Supplementary Table S3). At the team level, Distance Covered declined from Pre to During (*p* = 8.55 × 10^−4^) and then rebounded During to Post (*p* = 2.70 × 10^−5^). Workload showed no change from Pre to During (*p* > 0.05) but decreased During to Post (*p* = 0.024), and TRIMP likewise was stable Pre to During (*p* > 0.05) yet fell During to Post (*p* = 0.014). Within the position group, only Distance Covered decreased Pre to During (*p* = 0.032), with no significant shifts in Workload or TRIMP (*p* > 0.05). For the individual athlete, Distance Covered declined Pre to During (*p* < 0.001) and remained lower Pre to Post (*p* = 3.00 × 10^−4^), while Workload and TRIMP exhibited no significant differences (*p* > 0.05).

**Table 1:** Statistical analysis of player, position, and team data for beyond pulse, Whoop 4.0, and subjective data pre Ramadan, during Ramadan, and post Ramadan.

Metrics	Player			Position			Team		
	Pre-During	During-Post	Pre-Post	Pre-During	During-Post	Pre-Post	Pre-During	During-Post	Pre-Post
Beyond pulse metrics									
Distance (m)	No significant Difference			No significant Difference			8.55 × 10^−4^***	2.70 × 10^−5^***	1.00
Workload (A.U)	No significant Difference			No significant Difference			1.00	0.024*	0.409
TRIMP (A.U)	No significant Difference			No significant Difference			0.016*	0.014	1.00
WHOOP 4.0 metrics									
Strain (%)	No significant Difference			No significant Difference			1.00	1.01 × 10^−4^***	1.07 × 10^−4^***
Recovery (%)	No significant Difference			No significant Difference			No significant Difference		
RHR (bpm)	No significant Difference			No significant Difference			No significant Difference		
HRV (ms)	No significant Difference			No significant Difference			No significant Difference		
Sleep Performance (%)	No significant Difference			No significant Difference			4.00 × 10^−6^***	0.894	0.005**
Sleep Duration (min)	4.30 × 10^−3^***	0.501	3.00 × 10^−4^***	0.032*	0.916	0.029*	3.68 × 10^−7^***	1.00	2.77 × 10^−7^***
Subjective metrics									
RPE (6–20)	No significant Difference			No significant Difference			0.058	0.637	3.75 × 10^−3^***
Stress level (1–10)	1.00	0.028*	0.119	No significant Difference			2.00 × 10^−6^***	0.019	0.336
Muscle soreness (1–10)	No significant Difference			No significant Difference			1.01 × 10^−3^***	5.60 × 10^−5^***	0.889
Energy level (6–20)	No significant Difference			No significant Difference			5.80 × 10^−3^***	5.55 × 10^−4^***	1.00

* *p* < 0.05. **p < 0.01. ***p < 0.005.

**Figure 4:**
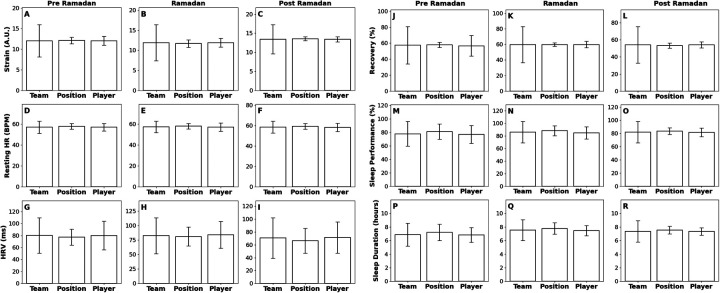
Bar plots showing the metrics recorded by Whoop 4.0—Strain (A.U.), Recovery (A.U.), Resting Heart Rate (bpm), Heart Rate Variability (ms), Sleep Performance (%), and Sleep Duration (h)—across three categories: Team, Position, and Player. Each row represents a different metric measured by the WHOOP wrist monitor. **(A–C)** Strain, **(D–F)** recovery, **(G–I)** resting heart rate, **(J–L)** heart rate variability, **(M–O)** sleep performance, and **(P–R)** sleep duration. Each box in the subfigure compares the metrics within different contexts: the first column compares metrics at the Team level, the second at the Position level, and the third at the Player level. Each subfigure in the row represents the metrics during a specific period: **(A,D,G,J,M,P)**: Pre-Ramadan, **(B,E,H,K,N,Q)**: Ramadan, and (**C,F,I,L,O,R)**: Post-Ramadan. Statistically significant differences are denoted by asterisks (*p < 0.05; **p < 0.01; ***p < 0.005) with effect sizes (r: non-parametric, d: parametric) denoted; comparisons without asterisks were not significant.

**Figure 5:**
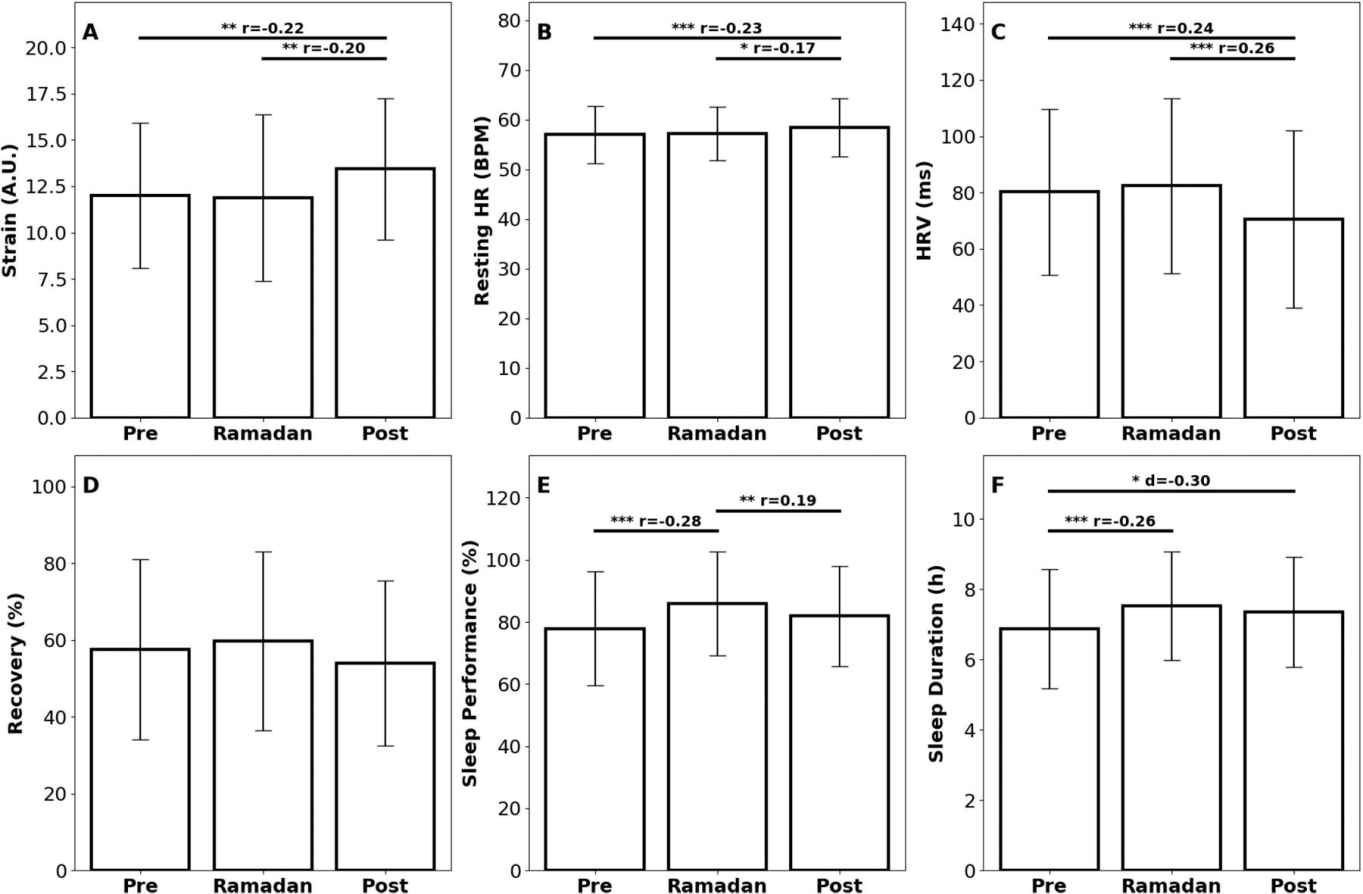
Bar plots displaying metrics collected by the Whoop wrist monitor for the individual player: across the three time periods (Pre-Ramadan, Ramadan, and Post-Ramadan). **(A)** Strain, **(B)** resting heart rate, **(C)** sleep performance, **(D)** recovery, **(E)** heart rate variability, and **(F)** Sleep Duration. Statistically significant differences are denoted by asterisks (*p < 0.05; **p < 0.01; ***p < 0.005) with effect sizes (*r*: non-parametric, *d*: parametric) denoted; comparisons without asterisks were not significant.

To assess the physiological changes experienced by the team, position group, and athlete, various metrics recorded by the Whoop 4.0 device were analyzed (17,662 data points) (Figures 4, 5 and Tables 1, 2). No significant changes observed in Strain, Recovery, Resting HR, or HRV at any level (*p* > 0.05). Team-level Sleep Performance decreased Pre to During (*p* = 4.00 × 10^−6^) and remained reduced Pre to Post (*p* = 0.005), with no significant changes at the position or individual level (*p* > 0.05). Sleep Duration at the team level fell by 14.6% Pre to During (*p* = 3.68 × 10^−7^) and remained lower Pre to Post (*p* = 2.77 × 10^−7^); the individual athlete's Sleep Duration also decreased (14.6%) Pre to During (*p* = 0.004) and Pre to Post (*p* = 3.00 × 10^−4^), whereas the position group showed no significant shifts (*p* > 0.05).

**Table 2:** Descriptive statistics of player, position, and team data for beyond pulse, Whoop 4.0, and subjective data pre Ramadan, during Ramadan, and post Ramadan.

Metrics	Player			Position			Team		
	Mean (St. dev.)			Mean (St. dev.)			Mean (St. dev.)		
	Pre	During	Post	Pre	During	Post	Pre	During	Post
Beyond pulse metrics									
Distance (m)	4,531 (1,903)	5,645 (2,562)	4,457 (2,562)	5,061 (1,867)	5,635 (2,897)	5,146 (2,867)	4,754 (1,882)	5,565 (2,624)	4,974 (2,439)
Workload (A.U)	10.67 (5.31)	11.6 (7.71)	9.54 (6.50)	11.7 (4.61)	12.0 (6.56)	11.7 (6.43)	11.0 (4.76)	11.8 (6.23)	11.0 (5.84)
TRIMP (A.U)	277 (81.3)	325 (147)	269 (129)	258 (72)	276 (126)	260 (114)	238 (77)	273 (107)	260 (104)
WHOOP 4.0 metrics									
Strain (%)	14.8 (4.54)	15.9 (5.03)	16.7 (3.76)	14.1 (4.13)	14.5 (4.13)	15.3 (3.78)	12.8 (4.11)	12.7 (4.53)	14.2 (3.72)
Recovery (%)	46.0 (28.7)	49.2 (23.6)	54.8 (24.0)	57.8 (24.6)	57.5 (19.1)	59.8 (20.3)	60.2 (21.8)	61.0 (22.1)	59.5 (21.1)
RHR (bpm)	60.4 (5.82)	60.9 (5.49)	58.7 (5.10)	54.9 (7.07)	54.3 (5.72)	53.9 (5.74)	56.5 (7.11)	57.2 (7.63)	57.3 (7.05)
HRV (ms)	73.9 (19.8)	70.3 (21.7)	66.4 (13.1)	69.8 (22.3)	73.8 (23.9)	71.1 (20.8)	89.7 (38.9)	89.1 (38.5)	86.1 (42.4)
Sleep performance (%)	55.0 (24.3)	67.3 (19.2)	64.3 (16.0)	66.8 (19.1)	71.0 (16.0)	70.4 (15.5)	75.8 (17.0)	80.5 (17.6)	79.9 (16.3)
Sleep duration (min)	263 (84)	361 (79.5)	394 (103)	370 (102)	409 (88.0)	415 (91.0)	402 (91.4)	430 (91.9)	437 (90.0)
Subjective metrics									
RPE (6–20)	12.8 (1.84)	13.7 (1.76)	12.7 (3.40)	13.4 (1.91)	13.9 (1.68)	13.2 (2.10)	14.0 (2.09)	13.6 (2.34)	13.5 (2.81)
Stress level (1–10)	4.71 (2.09)	4.84 (2.42)	3.22 (1.44)	4.00 (1.77)	3.65 (2.18)	3.28 (1.54)	4.20 (1.75)	3.70 (1.85)	3.96 (1.54)
Muscle soreness (1–10)	4.43 (1.50)	4.62 (1.68)	4.56 (1.50)	4.21 (1.57)	3.92 (1.65)	3.81 (1.55)	4.39 (1.84)	3.99 (1.79)	4.55 (1.85)
Energy level (6–20)	14.3 (1.07)	14.3 (1.73)	14.2 (1.42)	14.5 (1.27)	14.7 (2.30)	14.4 (1.76)	13.7 (2.43)	14.3 (2.30)	13.8 (2.17)

**Figure 6:**
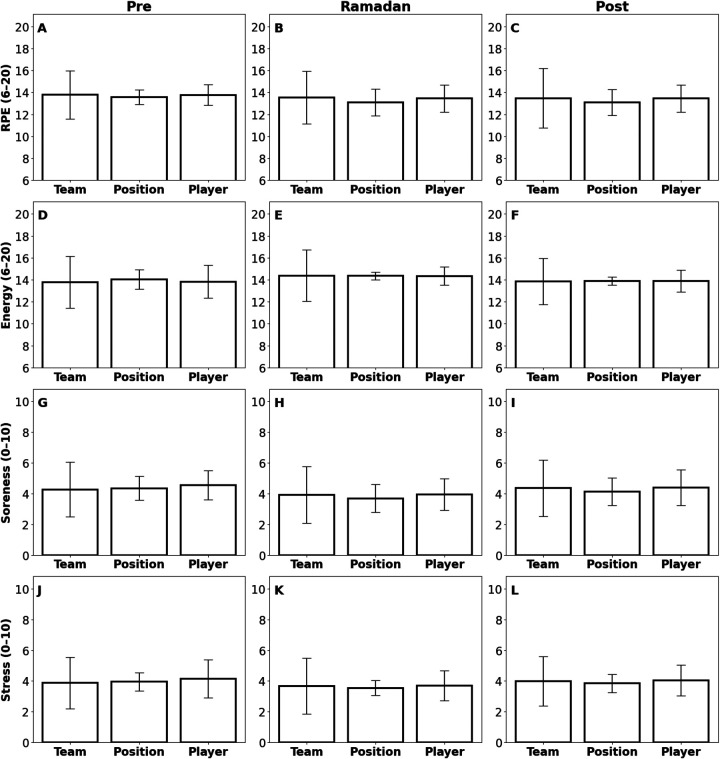
Bar plots illustrating the distribution of four subjective metrics—Rate of Perceived Exertion (RPE) (6–20 Scale), Energy (6–20 Scale), Soreness (1–10 Scale), and Stress (1–10 Scale)—across three analytical dimensions: Team, Position, and Player. Panels **(A–C)** display RPE data collected post-activity (training, lift, games). Panels **(D–F)** represent daily Energy levels, independent of specific activities. Panels G-I show daily Soreness metrics, and panels **(J–L)** present daily Stress levels, both regardless of specific activities. The columns of subfigures provide comparisons within different contexts: Team-level (first column), Position-level (second column), and Player-level (third column). Each row of subfigures corresponds to distinct periods: Pre-Ramadan **(A,D,G,J)**, Ramadan **(B,E,H,K)**, and Post-Ramadan **(C,F,I,L)**. Statistically significant differences are denoted by asterisks (*p < 0.05; **p < 0.01; ***p < 0.005) with effect sizes (*r*: non-parametric, *d*: parametric) denoted; comparisons without asterisks were not significant.

**Figure 7:**
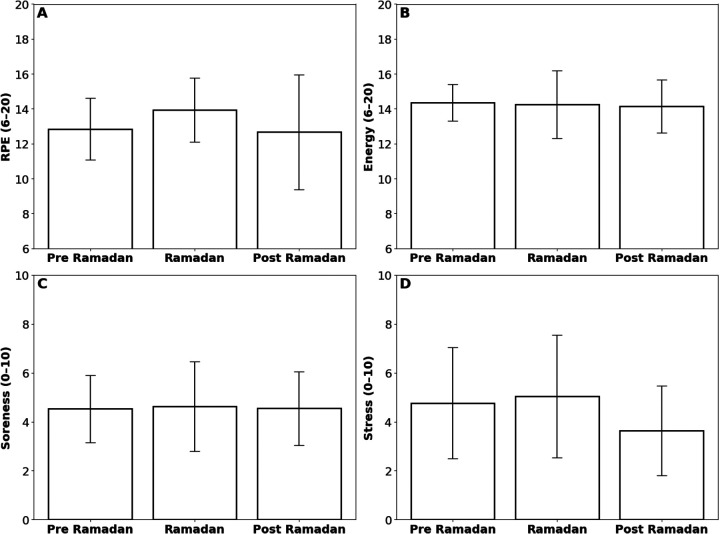
Bar plots displaying metrics collected by subjective self-reporting for the individual player across the three time periods: Pre-Ramadan, Ramadan, and Post-Ramadan. **(A)** Rating of Perceived Exertion (RPE, 6–20 scale), **(B)** Energy (6–20 scale), **(C)** Soreness (1–10 scale), and **(D)** Stress (1–10 Scale). Statistically significant differences are denoted by asterisks (*p < 0.05; **p < 0.01; ***p < 0.005) with effect sizes (*r*: non-parametric, *d*: parametric) denoted; comparisons without asterisks were not significant.

To complement the objective data, subjective metrics reported by the athlete were analyzed (12,921 data points) (Figures 6, 7; Tables 1 and 2). RPE did not change significantly at the team or positional level (*p* > 0.05), but the player RPE Pre to Post changed significantly (*p* = 3.75 × 10^−3^). Stress Levels at the team level rose Pre to During (*p* = 2.00 × 10^−6^) then fell During to Post (*p* = 0.019); the player experienced a significant During to Post reduction (*p* = 0.028), and no position-level effects were observed (*p* > 0.05). Muscle Soreness in the team increased Pre to During (*p* = 1.01 × 10^−3^) and decreased During to Post (*p* = 5.60 × 10^−5^), and Energy Levels followed a similar pattern (Pre to During: *p* = 5.80 × 10^−3^; During to Post: *p* = 5.55 × 10^−4^); position- and individual-level changes were non-significant (*p* > 0.05).

Additional analysis of the TEI across different periods, the individual athlete's TEI was compared with the TEI of the team and then positional group. Supplementary Figure S2 illustrates this comparison through time-series plots as well as discerning differences. Supplementary Figure S3 portrays histograms of each variable collected and analyzed in the study. Statistical analysis was performed and summarized in Table 2 showing the mean values and standard deviations for the performance data (Distance, Workload TRIMP), physiological data (Strain, Recovery, RHR, HRV, Sleep Performance, Sleep Duration), and subjective data (RPE, Stress Level, Muscle Soreness, Energy Level). Table 1 presents the results from statistical analysis across the Pre-Ramadan (Pre), Ramadan (During), and Post-Ramadan (Post) periods for Team, Position, and Player.

## 4 Discussion

This case study evaluated how Ramadan observance influenced performance, physiological regulation, and subjective perceptions in a female Division I collegiate athlete, benchmarked against positional and team-level data. Notable fluctuations were observed pre-, during-, and post-Ramadan monitoring periods. At the individual level, sleep duration declined markedly during Ramadan, while subjective stress, soreness, and reduced energy became prominent and persistent beyond Ramadan. At the team level, distance covered significantly decreased during Ramadan but then rebounded after, while workload and TRIMP also shifted. These fluctuations highlight the value of continuous multimodal monitoring to capture subtle phase-specific adaptations that yield real-time, actionable adjusts to athlete microcycle design during Ramadan, insights that intermittent testing would miss.

For the individual athlete, sleep duration and stress levels emerged as the most disrupted during Ramadan. Sleep duration declined during Ramadan and did not fully rebound post-Ramadan. Similarly, sleep performance declined across the Ramadan period. These findings align with Chamari et al. and Kerkeni et al., who attributed similar sleep disruptions to late-night prayers (Taraweeh) and early morning meals (Suhoor) (18, 19). Alghamdi et al. similarly reported reduced sleep in individuals, supporting the utility of wearables for continuous monitoring (20). The athlete also reported heightened stress and soreness during Ramadan, with heightened stress continued post-Ramadan. Energy levels also declined during Ramadan, indicating accumulated fatigue. These trends mirror Lipert et al., who reported elevated psychological strain in observing athletes (2), likely related to disrupted recovery cycles, altered sleep, and reduced caloric intake (not studied in this work). Despite these stressors, no significant changes were observed in other physiological metrics such as HRV or recovery percentage, suggesting potential individual resilience or effective self-regulation of external load during Ramadan when sleep debt persists.

At the positional group level, the only notable difference was reduced sleep duration during Ramadan. This contrasts with the individual athlete's data, suggesting that group-level sleep may be more influenced by shared external factors, such as varying academic workloads, team responsibilities, or social dynamics. These findings underscore the importance of considering external commitments and group dynamics when evaluating Ramadan-related effects on sleep and recovery, as well as when assessing athletes on an individual basis, which allows for benchmarking each observing athlete against rolling position-level norms and then tailoring support accordingly rather than attributing deviations solely to fasting.

At the team level, multiple performance metrics shifted across the Ramadan timeline. Distance covered decreased during Ramadan and rebounded afterwards, while workload, and TRIMP also shifted, likely due to structured training periodization. These trends align with Chaouachi et al. and Aziz et al., who reported performance stabilization or rebound after initial decrements during Ramadan fasting in elite judo and endurance athletes, respectively (5, 14). In this study however, the fluctuations likely reflect changes in training periodization rather than the direct effects of Ramadan observation. Importantly, while absolute changes were observed, the athlete's TEI remined comparable to positional and team data, indicating preserved internal–external load coupling and potential adaptation. This reinforces the value of individualized, continuous monitoring in identifying compensatory strategies or resilience, which might not be apparent through traditional intermittent testing. Operationally, use metrics to guide dose–response control with an upward deviation from the athlete's rolling baseline (e.g., >∼1 SD) denotes decoupling and warrants intervention; and within expected error supports continued progression.

This study underscores the intricate relationship between religious observance, athletic performance, and external pressures such as academic and social demands. While the individual athlete demonstrated distinct changes, group-level patterns reflect the broader impact of environmental and contextual factors, including fluctuating training schedules and recovery constraints. These findings highlight the importance of considering both individual and collective adaptations when assessing athletic performance to gain important individualized context on data fluctuations. The integration of wearable technologies provided a level of precision in monitoring physiological, biomechanical, and subjective metrics, offering critical insights into how alterations in routine, such as observing Ramadan, impacts performance and recovery by distinguishing between observance related perturbations (e.g., sleep disruption and elevated perceived exertion) from training periodization effects. Continuous objective monitoring through tools such as Beyond Pulse and Whoop 4.0 allowed for real-time tracking of key metrics, including workload, HR, RHR, HRV, sleep performance, and recovery. This approach not only identified fluctuations in metrics at both individual and group level but also provided actionable data to tailor interventions for both the individual athlete and the team. Operationally, multimodal monitoring lets staff quickly adjust session density, volume, and timing for each athlete, using position and team trends as benchmarks, to keep workload and physiological response in balance.

This study has limitations. Firstly, the sample size was restricted to a single Division I female soccer athlete, which limits the generalizability of findings to other sports, genders, or competitive levels. While comparisons with positional and team averages offered some external context outside of observation, they do not fully account for individual variability. Secondly, external influences such as academic stressors, social commitments, and team dynamics were not controlled, which may have confounded the observed changes in sleep, recovery, and performance metrics. Since only one athlete observed Ramadan, positional and team-level results may reflect broader environmental changes rather than the specific effects of observation. Although wearable devices provide continuous and objective monitoring, the accuracy of the data acquired may be limited by several factors. Potential sources of error include participant non-compliance (inconsistent or incorrect device wear) and intrinsic hardware constraints. Additional influences such as sensor sensitivity, battery capacity, environmental conditions, and synchronization fidelity may further compromise data integrity. Moreover, reliance on subjective metrics introduces inherent variability, as self-reported measures are susceptible to personal and contextual biases. Finally, this study did not include direct assessments of nutritional intake or hydration status, which may provide additional context for interpreting physiological responses specifically when fasting.

Future studies should not only address these limitations but also expand in several key directions. First, longitudinal studies involving larger, more diverse athlete cohort-including both male and female participants across various sports are needed to improve the generalizability of the findings. Secondly, integrating biochemical markers such as cortisol, glucose, and ketone levels alongside wearable data could offer deeper insight into physiological adaptations during Ramadan and broaden the interpretation of observed changes. Third, future studies should investigate the effect of targeted interventions—including modified training schedules, optimized recovery protocols, and tailored nutritional strategies—on athletes performance and well-being during Ramadan. Although this study highlights the potential of continuous monitoring to evaluate lifestyle changes, such as Ramadan, addressing these limitations and future research directions can strengthen the findings and provide more robust conclusions. This will enable the development of generalizable, evidence-based recommendations to support athletes in optimizing performance and recovery during periods of significant lifestyle adjustments.

## 5 Conclusion

This case study seeks to highlight the need for continuous monitoring of athletes leveraging wearable technologies to elucidate a holistic profile, as continuous data provides more sensitive data collection. It addresses the intricacies of individuals participating in sport notably the impact of religious observation, such as Ramadan, on performance. Continuous, multimodal monitoring revealed phase-specific responses during observation in a single Division I collegiate athlete, benchmarked against the team and position group during the Spring 2024 season. During Ramadan, sleep duration decreased by 14.6% (*p* = 3.68 × 10^−7^), perceived energy was lower (*p* = 5.80 × 10^−3^), and subjective stress remined elevated post-Ramadan (*p* = 0.028). In contrast, physiological indices (HRV, HR, recovery) and load coupling (TEi) were stable relative to position and team benchmarks, indicating preserved coupling and adaptive regulation despite altered routines.

Integrating a multimodal approach of wearable physiological monitors, external load monitor, and subjective questionnaires allowed observation-related perturbations to be distinguished from training-periodization effects and supported timely, individualized adjustments to session density, volume, and timing. While bounded by a single-case design, this feasible, low-burden monitoring approach provides clear, actionable control of the dose–response relationship during Ramadan without compromising planned training. Thus, multimodal approaches can assist coaches and athletes in making informed decisions about training loads, nutritional intake, and recovery strategies, ensuring optimal performance, and maintaining health with potential to shift how athletes prepare for and navigate various external factors, like Ramadan, enhancing their overall well-being and competitive success.

## Data Availability

The data and datasets used in this article are not avaiable due to privacy restrictions. Any requests please contact the corresponding author, Dhruv Seshadri, dhs223@lehigh.edu.

## References

[1] Abaïdia AE Daab W Bouzid MA. Effects of Ramadan fasting on physical performance: a systematic review with meta-analysis. Sports Med. (2020) 50(5):1009–26. 10.1007/s40279-020-01257-0 31960369

[2] Lipert A Kozłowski R Rasmus P Marczak M Timler M Timler D Sleep quality and performance in professional athletes fasting during the month of Ramadan. Int J Environ Res Public Health. (2021) 18(13):6890. 10.3390/ijerph18136890 34198990 PMC8295756

[3] Memari AH Kordi R Panahi N Nikookar LR Abdollahi M Akbarnejad A. Effect of Ramadan fasting on body composition and physical performance in female athletes. Asian J Sports Med. (2011) 2(3):161–6. 10.5812/asjsm.34754 22375235 PMC3289211

[4] Anis C Leiper JB Nizar S Coutts AJ Karim C. Effects of Ramadan intermittent fasting on sports performance and training: a review. Int J Sports Physiol Perform. (2009) 4(4):419–34. 10.1123/ijspp.4.4.419 20029094

[5] Aziz AR Wahid MF Png W Jesuvadian CV. Effects of Ramadan fasting on 60 min of endurance running performance in moderately trained men. Br J Sports Med. (2010) 44(7):516–21. 10.1136/bjsm.2009.070425 20519256

[6] Shephard RJ. Ramadan and sport: minimizing effects upon the observant athlete. Sports Med. (2013) 43(12):1217–41. 10.1007/s40279-013-0080-7 23888431

[7] Mhenni T Souissi A Tayech A Yousfi N Mejri MA Chamari K The effect of Ramadan fasting on the morning–evening difference in team-handball-related short-term maximal physical performances in elite female team-handball players. Chronobiol Int. (2021) 38(10):1488–99. 10.1080/07420528.2021.1932994 34112026

[8] Adesida Y Papi E McGregor AH. Exploring the role of wearable technology in sport kinematics and kinetics: a systematic review. Sensors. (2019) 19(7):1597. 10.3390/s19071597 30987014 PMC6480145

[9] Zerguini Y Kirkendall D Junge A Dvorak J. Impact of Ramadan on physical performance in professional soccer players. Br J Sports Med. (2007) 41(6):398–400. 10.1136/bjsm.2006.032037 17224435 PMC2465333

[10] Saw AE Main LC Gastin PB. Monitoring the athlete training response: subjective self-reported measures trump commonly used objective measures: a systematic review. Br J Sports Med. (2016) 50(5):281–91. 10.1136/bjsports-2015-094758 26423706 PMC4789708

[11] Amjad M Cavallario JM Harris NA Welch Bacon CE. Muslim collegiate student-Athletes’ experience with fasting during Ramadan while participating in sport. J Athl Train. (2024) 59(5):474–86. 10.4085/1062-6050-0363.23 38014802 PMC11127679

[12] El-Khatib AH Tolbert TA McIlvain GE. Participation of muslim athletes during the month of Ramadan. Int J Athl Ther Train. (2012) 17(5):41–5. 10.1123/ijatt.17.5.41

[13] Hamouda O Chtourou H Farjallah MA Davenne D Souissi N. The effect of Ramadan fasting on the diurnal variations in aerobic and anaerobic performances in Tunisian youth soccer players. Biol Rhythm Res. (2012) 43(2):177–90. 10.1080/09291016.2011.560050

[14] Chaouachi A Coutts AJ Chamari K Wong DP Chaouachi M Chtara M Effect of Ramadan intermittent fasting on aerobic and anaerobic performance and perception of fatigue in male elite judo athletes. J Strength Cond Res. (2009) 23(9):2702–9. 10.1519/JSC.0b013e3181bc17fc 19910805

[15] Brisswalter J Bouhlel E Falola JM Abbiss CR Vallier JM Hauswirth C. Effects of Ramadan intermittent fasting on middle-distance running performance in well-trained runners. Clin J Sport Med. (2011) 21(5):422–7. 10.1097/JSM.0b013e3182293891 21857506

[16] Bouhlel E Salhi Z Bouhlel H Mdella S Amamou A Zaouali M Effect of Ramadan fasting on fuel oxidation during exercise in trained male rugby players. Diabetes Metab. (2006) 32(6):617–24. 10.1016/S1262-3636(07)70317-8 17296516

[17] Trabelsi K Ammar A Boukhris O Boujelbane MA Clark C Romdhani M Ramadan intermittent fasting and its association with health-related indices and exercise test performance in athletes and physically active individuals: an overview of systematic reviews. Br J Sports Med. (2024) 58(3):136–43. 10.1136/bjsports-2023-106826 37923379

[18] Chamari K Haddad M Wong DP Dellal A Chaouachi A. Injury rates in professional soccer players during Ramadan. J Sports Sci. (2012) 30(Suppl 1):S93–102. 10.1080/02640414.2012.696674 22697802

[19] Kerkeni M Trabelsi K Kerkeni M Boukhris O Ammar A Salem A Ramadan fasting observance is associated with decreased sleep duration, increased daytime sleepiness and insomnia symptoms among student-athletes. Sleep Med. (2024) 122:185–91. 10.1016/j.sleep.2024.08.012 39182275

[20] Alghamdi AS Alghamdi KA Jenkins RO Alghamdi MN Haris PI. Impact of Ramadan on physical activity and sleeping patterns in individuals with type 2 diabetes: the first study using fitbit device. Diabetes Ther. (2020) 11(6):1331–46. 10.1007/s13300-020-00825-x 32367477 PMC7261298

[21] Aroganam G Manivannan N Harrison D. Review on wearable technology sensors used in consumer sport applications. Sensors. (2019) 19(9):1983. 10.3390/s19091983 31035333 PMC6540270

[22] Seçkin AÇ Ateş B Seçkin M. Review on wearable technology in sports: concepts, challenges and opportunities. Appl Sci. (2023) 13(18):10399. 10.3390/app131810399

[23] Ju F Wang Y Yin B Zhao M Zhang Y Gong Y Microfluidic wearable devices for sports applications. Micromachines (Basel). (2023) 14(9):1792. 10.3390/mi14091792 37763955 PMC10535163

[24] Benson LC Räisänen AM Volkova VG Pasanen K Emery CA. Workload a-WEAR-ness: monitoring workload in team sports with wearable technology. A scoping review. J Orthop Sports Phys Ther. (2020) 50(10):549–63. 10.2519/jospt.2020.9753 32998615

[25] Zadeh A Taylor D Bertsos M Tillman T Nosoudi N Bruce S. Predicting sports injuries with wearable technology and data analysis. Inf Syst Front. (2021) 23(4):1023–37. 10.1007/s10796-020-10018-3

[26] Jiang Z Hao Y Jin N Li Y. A systematic review of the relationship between workload and injury risk of professional male soccer players. Int J Environ Res Public Health. (2022) 19(20):13237. 10.3390/ijerph192013237 36293817 PMC9602492

[27] Andersen MB Williams JM. A model of stress and athletic injury: prediction and prevention. J Sport Exerc Psychol. (1988) 10(3):294–306. 10.1123/jsep.10.3.294

[28] Temm DA Standing RJ Best R. Training, wellbeing and recovery load monitoring in female youth athletes. Int J Environ Res Public Health. (2022) 19(18):11463. 10.3390/ijerph191811463 36141735 PMC9517577

[29] Rebelo A Martinho DV Valente-dos-Santos J Coelho-e-Silva MJ Teixeira DS. From data to action: a scoping review of wearable technologies and biomechanical assessments informing injury prevention strategies in sport. BMC Sports Sci Med Rehabil. (2023) 15(1):169. 10.1186/s13102-023-00783-4 38098071 PMC10722675

[30] Montull L Slapšinskaitė-Dackevičienė A Kiely J Hristovski R Balagué N. Integrative proposals of sports monitoring: subjective outperforms objective monitoring. Sports Med Open. (2022) 8(1):41. 10.1186/s40798-022-00432-z 35348932 PMC8964908

[31] Delaney JA Duthie GM Thornton HR Pyne DB. Quantifying the relationship between internal and external work in team sports: development of a novel training efficiency index. Sci Med Footb. (2018) 2(2):149–56. 10.1080/24733938.2018.1432885

