# Supplementary material for: A case study on the impact of Ramadan on biomechanical and physiological markers in a female collegiate student-athlete
Source: Front Sports Act Living. 2025 Oct 21;7:1576424. doi: 10.3389/fspor.2025.1576424 (PMC12583904; doi:10.3389/fspor.2025.1576424)
Supplement: Supplementary file 1 [file Table1.docx]

| **Source** | **Variable** | **Unit** |
| --- | --- | --- |
| Beyond Pulse | Distance Covered | meters |
|  | HR | bpm |
|  | Workload | A.U. |
|  | TRIMP (Training Impulse) | A.U. |
| Whoop 4.0 | Strain | A.U. |
|  | Recovery | % |
|  | RHR | bpm |
|  | HRV | ms |
|  | Sleep Performance | % |
|  | Sleep Duration | hours |
| Questionnaire | RPE (Rate of Perceived Exertion) | 6–20 |
|  | Stress Level | 1–10 |
|  | Muscle Soreness | 6–20 |
|  | Energy Level | 6–20 |
| Derived | TEI (Training Efficiency Index) | Ratio |

**Table S1: List of Variables and Their Respective Sources With Units**
