# Supplementary material for: A case study on the impact of Ramadan on biomechanical and physiological markers in a female collegiate student-athlete
Source: Front Sports Act Living. 2025 Oct 21;7:1576424. doi: 10.3389/fspor.2025.1576424 (PMC12583904; doi:10.3389/fspor.2025.1576424)
Supplement: Supplementary file 2 [file Table2.docx]

| **Question** | **Metric** | **Explanation** |
| --- | --- | --- |
| Name | - | Identify Player |
| Energy Level | 6-20 | Numerically assess energy level |
| Muscle Soreness | 0-10 | Numerically assessing muscle soreness |
| Location | - | Identify location of soreness |
| Stress | 0-10 | Numerically assess stressors |
| Hours of Sleep | - | Identify hours of sleep the player got |
| Sleep Quality | Very Restless,Restless, Restful, Very Restful | Identify the quality of sleep the player got |
| Nap Length | 0-10 | Numerically identify nap duration, if one was taken |
| Illness/Injury | - | Identify any illness or injury to report |
| Did you utilize sports medicine | - | Identify if sports medicine/athletic trainer was used/seen |
| Whoop Recovery | 0-100% | Identify Whoop Recovery Score, compared against the score from Whoop Unite |
| Are you on your period | Yes/No | Identify if the player is menstruating |
| Menstrual cycle related symptoms | - | Identify if the player is experiencing any menstrual cycle related symptoms |

**Table S2: Questions and Relative Metrics for Daily Readiness Questionnaire**
