# Supplementary material for: A case study on the impact of Ramadan on biomechanical and physiological markers in a female collegiate student-athlete
Source: Front Sports Act Living. 2025 Oct 21;7:1576424. doi: 10.3389/fspor.2025.1576424 (PMC12583904; doi:10.3389/fspor.2025.1576424)
Supplement: Supplementary file 3 [file Table3.docx]

| **Question** | **Metric** | **Explanation** |
| --- | --- | --- |
| Name | - | Identify Player |
| Session Type | Training, Lift, Game,  Recovery, Individual | Assess the type of Activity |
| Duration | Minutes | Assess duration of activity |
| RPE | Borg Scale (6-20) | Identify perceived exertion of the activity |

**Table S3: Question and Relative Metrics for Post-Session Questionnaire**
