# Supplementary figures and images for: A case study on the impact of Ramadan on biomechanical and physiological markers in a female collegiate student-athlete
Source: Front Sports Act Living. 2025 Oct 21;7:1576424. doi: 10.3389/fspor.2025.1576424 (PMC12583904; doi:10.3389/fspor.2025.1576424)

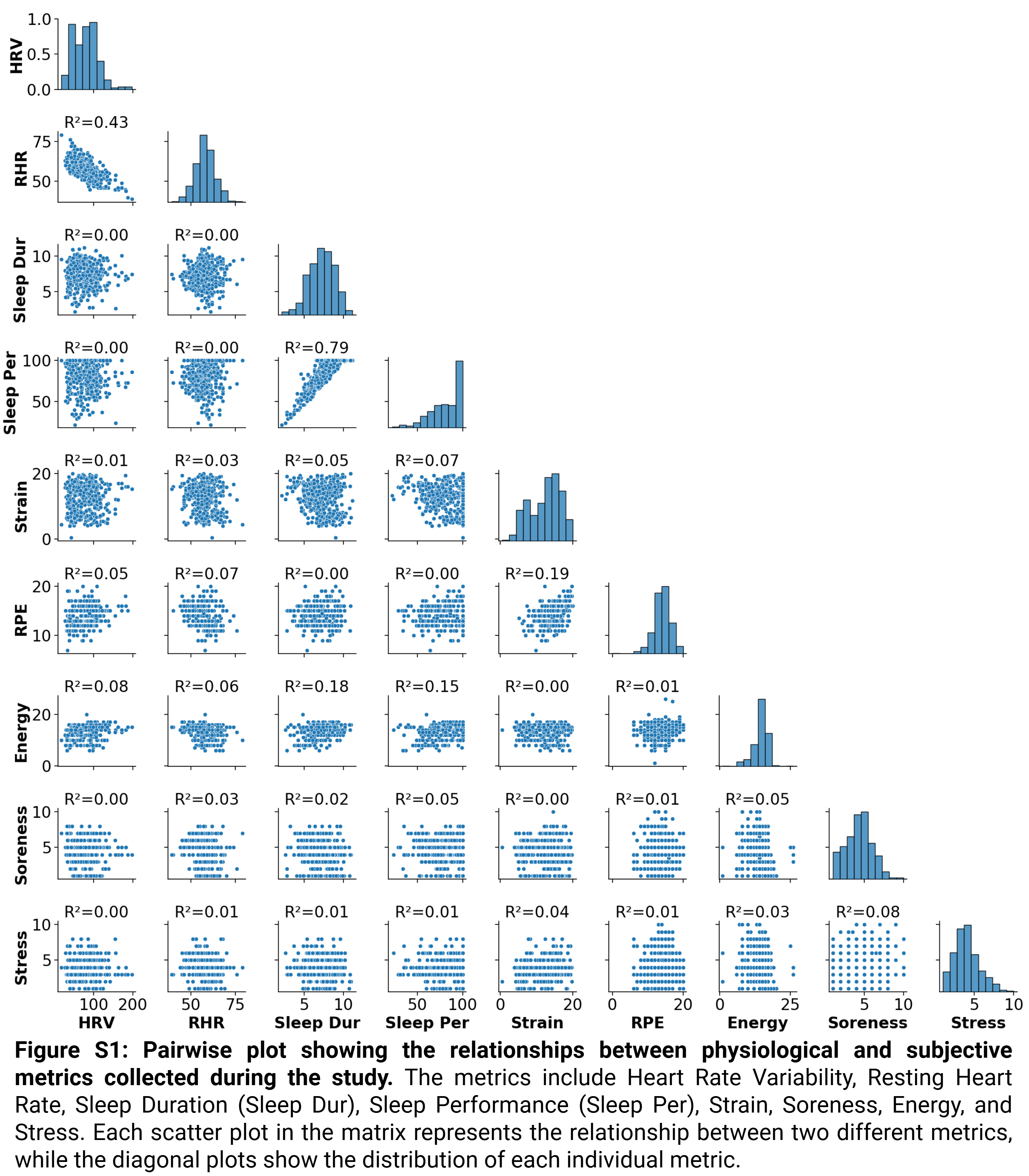

Supplement: Supplementary file 4 [file Image1.png]

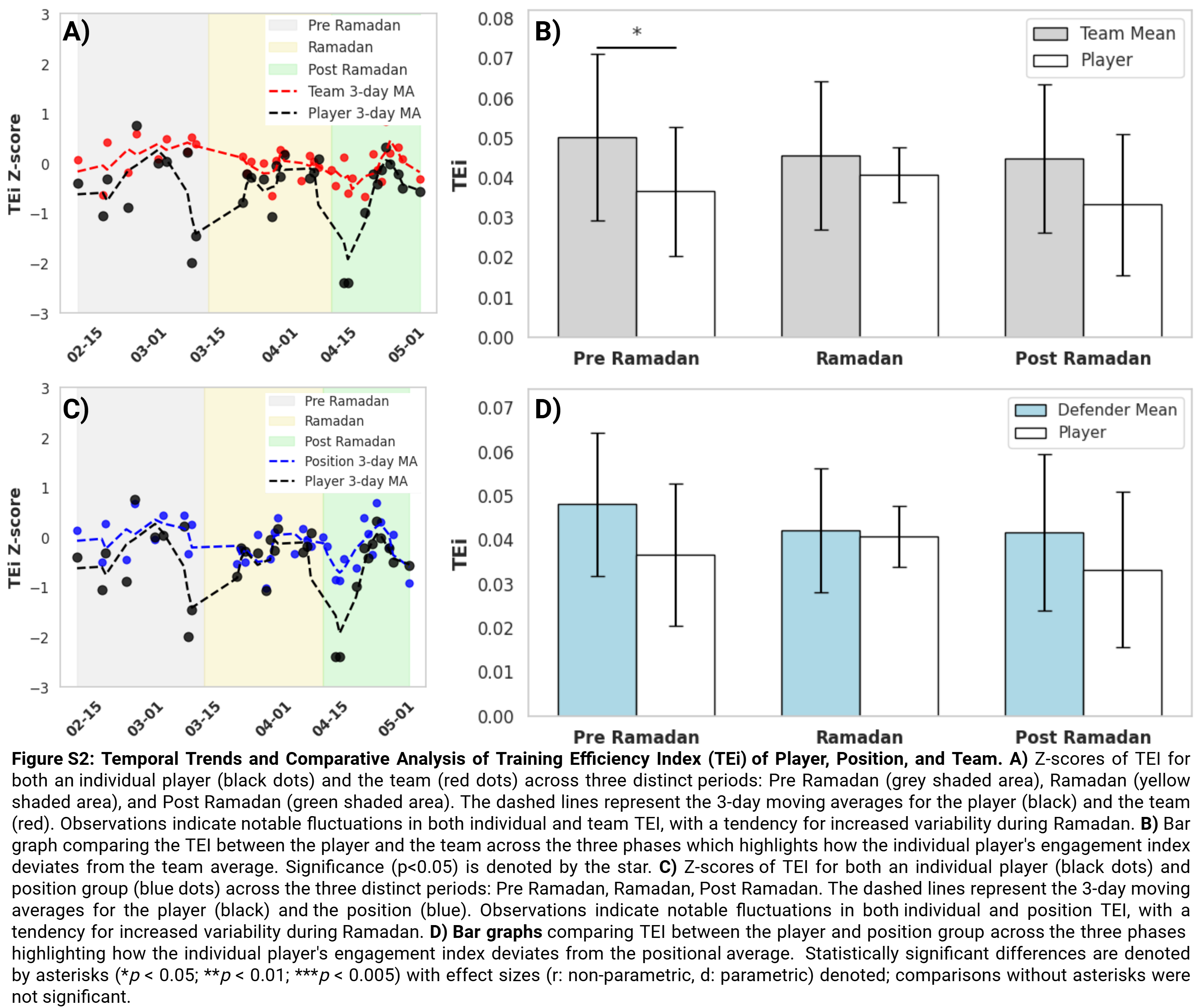

Supplement: Supplementary file 5 [file Image2.png]

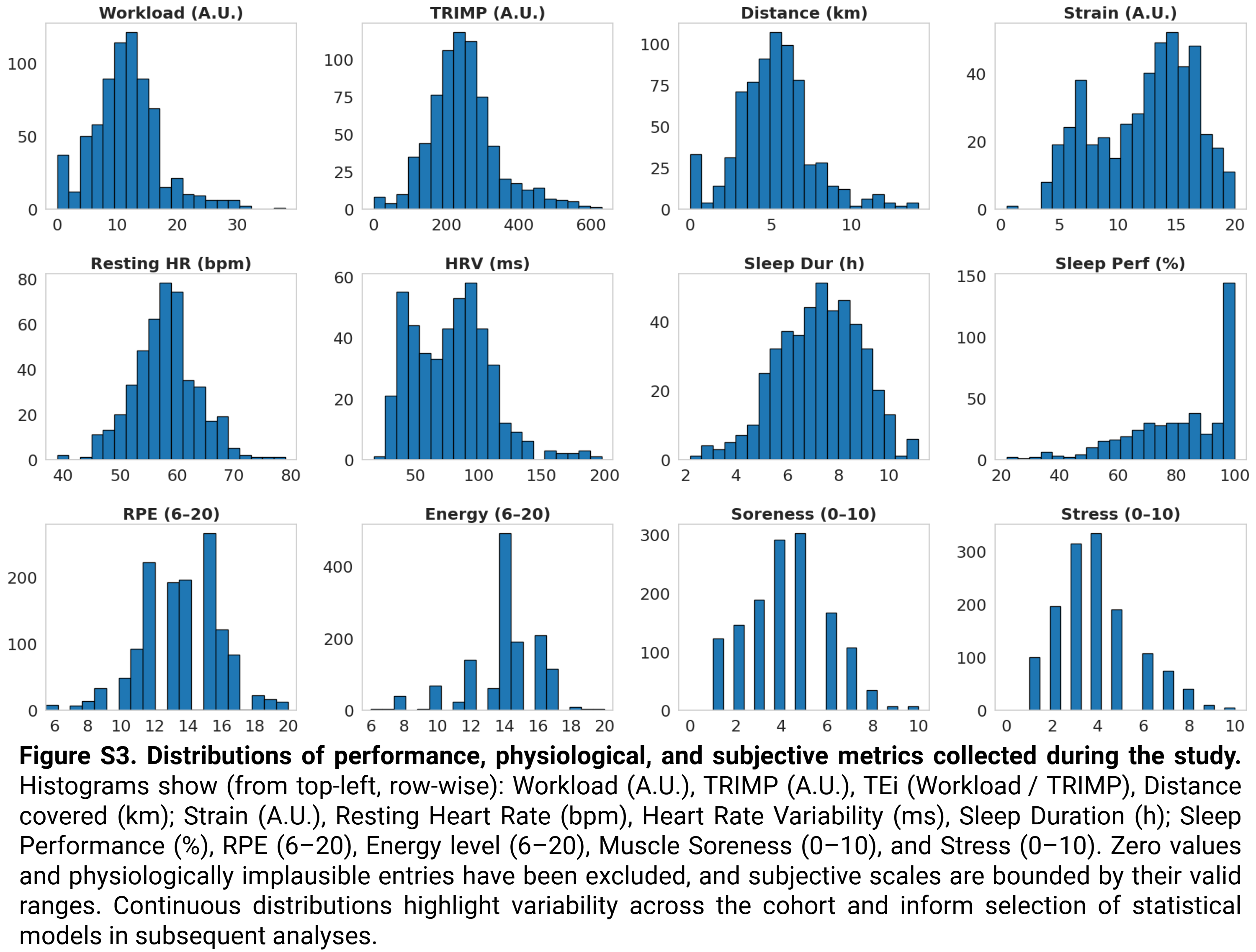

Supplement: Supplementary file 6 [file Image3.png]
